# Chemistry, Pharmacy, &c.

**Published:** 1836-07

**Authors:** 


### CHEMISTRY, PHARMACY, &c.

### *Detection of Sugar in the Blood in Diabetes, and on the best Mode of separating the same substance from the Urine.* By Felice Ambrosioni, of Milan.

[It is well known that until very lately the most distinguished British and foreign chemists have failed to detect the presence of sugar in the blood of diabetic patients. This, however, has been recently effected by the author of the memoir of which we shall give an extract, and also, we believe, by some other continental chemists. If this does not prove to be only an exception to the general rule,—and we confess we are sceptical on the point, on considering that such eminent men as Guendeville, Nicolas, Wollaston, Henry, Marcet, Prout, failed to detect any trace of sugar in their experiments,—the discovery will occasion a great change in our views respecting the pathology of diabetes. It will lead to the conclusion, that in diabetes, as in other diseases, the office of the kidneys is chiefly to act as filters to the blood, by removing effete and unassimilated matters; also that the disorder does not reside in these organs, and consequently that our remedies must be directed to the improvement of the other functions. The following are Signor Ambrosioni's processes for detecting the saccharine principle in the blood and urine. It is proper to observe, that the discovery was made only in a single case; and it may not be uninteresting to mention that the case was cured by the use of creosote.]

#### 1. *Analysis of the Blood.*

To obtain sugar from the blood, one pound of the serum and crassamentum from the same patient was diluted with water boiled and filtered. The filtered liquor was deprived of its colour by subacetate of lead, and a stream of sulphuretted hydrogen threw down from the mixture a black pultaceous mass, which diluted with distilled water, and filtered, produced a dark brown liquor, which was boiled in a solution of albumen. As the albumen coagulated, the liquor separated into a clear colourless fluid and a dark flocculent and insoluble matter. The fluid portion, when boiled slowly, threw up a syrupy scum, and assumed all the characters of a perfect syrup. Left at rest for a few weeks, small colourless crystals were formed in it of a prismatic form, with a rhomboidal base, modified at the angles and apices, in every respect resembling a perfect sugar. The uncrystallized syrup, at about 80° F., mixed with a little yeast, exhibited a decided vinous fermentation. The quantity of syrup obtained from one pound of blood was about an ounce, the crystals weighed two grains. [Since the foregoing was transcribed for the press, a case has been published in the Medical Gazette, No. 431, in which sugar was detected in the blood by Mr. Maitland.]

II. *Analysis of the Urine.*

The process employed in the reduction of sugar from the urine is as follows: The urine, which was pale, void of urea, and contained few salts, was treated with subacetate of lead, the oxyde of which was precipitated, combined with the animal and colouring matters, and a few salts. Sulphuretted hydrogen, passed through the filtered liquor, removed the excess of the salts of lead, which formed a brown precipitate, the liquor remaining clear, and consisting of water, sugar, and a minute portion of animal matter. Evaporated in a water bath to a syrup, and left for some weeks at the ordinary temperature, a white efflorescence beginning at the sides of the vessel, gradually extends over the whole, and converts it into a solid, whitish, amorphous mass, which, when broken, exhibits indistinct traces of crystallization; and, when washed with pure alcohol, is freed from the animal and colouring matters, and remains undissolved in the form of a pure white sugar.—*Annali Universali di Medicina. Aprile,* 1835.

---

*On the Effects of Boiling Meat and Vegetables.* By M. Chevreul.

[In a report read before the Academy of Sciences, by M. Chevreul, who was appointed with others to examine the nature of soup sold in Paris under the name of "bouillon de la Compagnie Hollandaise," the results of experiments on boiling food in different ways are introduced, which convey some important dietetic hints.]

If meat is boiled in distilled water in which 1,125 of its weight of common salt is dissolved, it will be more tender and have more taste, whilst the decoction will be equally more sapid and more odorous, than if the well-water of Paris, which contains sulphate and carbonate of lime, had been employed. The sulphate of lime exercises a singularly unfavorable influence on the tenderness and savour of the meat, and odour and savour of the broth. If, however, the distilled water is saturated with common salt, the meat becomes more hard, with a particular taste analogous to ham, and the broth less odorous and sapid.

The mode of boiling influences the results. Thus, two pieces of meat of equal size, &c. were chosen: one was put in an earthen pot with a given quantity of cold distilled water, and the temperature gradually raised to ebullition, and maintained at the boiling point for five hours; the other was plunged into the same quantity of boiling water, and boiled also for five hours. The taste of the broth made from the meat placed in boiling water was unanimously pronounced, by ten persons, to be inferior to that made from the meat placed first in cold water; and, on concentrating and analyzing the former, it furnished only 10,1000 organic matter and 2,1000 fixed salts; whilst the latter furnished 13,1000 organic matter, and 3,1000 fixed salts. On the other hand, 500 grains of meat boiled gradually was reduced to 326 grains, and to 3 gr. 25 of fat which could be separated; whilst 500 grains of the meat plunged at once into the boiling water was only diminished to 375 grains, containing almost all the fat. This is owing to the albumen and fibrine of the outer surface being hardened immediately by the sudden heat before they could be dissolved, and thus forming a sort of coating which opposed the free penetration of the water into the interior of the meat. During the ordinary boiling of meat, the albumen is dissolved before the temperature of the water is elevated to that degree at which the albumen coagulates; when the temperature is sufficiently elevated, all the albumen is cooked, and is reduced partly to an insoluble solid, coloured slightly with hematosine which forms the scum, and partly to a soluble portion which remains dissolved in the water. The cellular tissue, which penetrates all parts of the meat, and particularly that which envelopes the fat, together with the tendinous tissue, is changed into two parts: one, which consists of gelatin, dissolves; the other remains in a solid state, more or less soft and swollen, and is commonly called the nerve. The muscular tissue, essentially composed of fibrine, becomes at first hard, like the albumen, but none of it dissolves; so that, if albumen, gelatinous tissue, stearine, and oleine, were not interposed between its parietes, the muscular tissue would be too stringy to be thought highly of as nutriment. The fat, formed of oleine and stearine, is not changed; one part remains in the meat, and another swims on the broth. The principle which imparts the predominant odour to the meat, the sulphurous product, the amber-smelling principle, (an unnamed acid, more or less analogous to hircic and butyric acids;) the volatile acid, analogous to acetic acid; all of which are obtained by the distillation of meat, appear to be formed by a new state of equilibrium, which is established between the elements of one or more principles immediately soluble in water.

The changes produced in boiling some vegetables are worth attending to. From the red cabbage, and probably all its varieties, when boiled in distilled water, is disengaged an odorous principle proper to many Cruciferæ, which blackens paper impregnated with acetate of lead, from its containing sulphur; and some ammonia. The turnip and parsnip give out a similar principle, but containing less sulphur. The burnt onion exhales a volatile oil, containing more sulphur than the volatile principle of the cabbage, and some ammonia. From the carrot is disengaged a strongly odoriferous principle, accompanied by ammonia, but not acting on acetate of lead. The same vegetables, boiled in distilled water, containing 1,125 of its weight of common salt, exhale the same products, but the odour of the carrot smells sweeter, and that of the Cruciferæ stronger. The pure water in which these vegetables have been boiled retains in solution a sensible quantity of the odoriferous principles of the carrot, onion, and parsnip, as well as the colouring matter, &c. The salted decoction smelled more strongly than the other; its taste was also stronger, (not taking into account the taste given by the salt,) and yet it contained less extractive matters, in the proportion of 1 to 1.4. It may therefore be concluded, that the salt develops and increases the sapidity of the matter extracted. The vegetables themselves are much more tender and sapid when boiled in water thus salted than in pure water. The onion, when boiled in pure water, is inodorous and insipid; when boiled in salted water, it has a sweet taste and marked aroma. Water, therefore, containing 1,125 of its weight of muriate of soda is more proper than pure water for boiling these vegetables: first, because the addition of salt renders the water less capable of taking up the soluble matters; and, secondly, because it gives the vegetables more tenderness, odour, and taste.

*Journal de Pharmacie, &c. Mai,* 1835.

---

*Simplified Method of obtaining Creosote.* By Ant. Giordano.

This substance, obtained by the new process, has exactly the same properties as that discovered by Reichenbach. The following is the mode of procuring it: distil wood-tar from the willow, at an elevated temperature from a tinned copper retort, until the residue has the consistence of a soft pitch. Re-distil the liquor passed over till its residue resembles the former. The liquor neutralized by subcarbonate of potass or lime-water is redistilled till all the oil of creosote has passed over. The oil is dissolved in caustic potass, from which, after simmering a little in a porcelain vessel, and cooling it, the Eupione which floats is easily separated. The same operation is repeated with the Eupione, to remove all the oil that is united to it. The saponaceous liquor, treated with diluted sulphuric acid, is distilled into water, from which the creosote is separated, and the water saturated with creosote is kept for external use, or redistilled for a concentrated acetic acid of a pungent and most agreeable odour.—*Annali di Medicina. Aprile,* 1835.

